# RNA viral vectors for improved *Agrobacterium*-mediated transient expression of heterologous proteins in *Nicotiana benthamiana* cell suspensions and hairy roots

**DOI:** 10.1186/1472-6750-12-21

**Published:** 2012-05-06

**Authors:** Jeffrey S Larsen, Wayne R Curtis

**Affiliations:** 1 Department of Chemical Engineering, The Pennsylvania State University, University Park, Pennsylvania, 16802, USA

**Keywords:** Plant tissue culture, Gene silencing, Viral vectors, Hairy roots, Plant cell suspensions, Potato virus X, Tobacco rattle virus, Transient protein expression, *Agrobacterium tumefaciens*

## Abstract

**Background:**

Plant cell suspensions and hairy root cultures represent scalable protein expression platforms. Low protein product titers have thus far limited the application of transient protein expression in these hosts. The objective of this work was to overcome this limitation by harnessing *A. tumefaciens* to deliver replicating and non-replicating RNA viral vectors in plant tissue co-cultures.

**Results:**

Replicating vectors derived from *Potato virus X* (PVX) and *Tobacco rattle virus* (TRV) were modified to contain the reporter gene β-glucuronidase (GUS) with a plant intron to prevent bacterial expression. In cell suspensions, a minimal PVX vector retaining only the viral RNA polymerase gene yielded 6.6-fold more GUS than an analogous full-length PVX vector. Transient co-expression of the minimal PVX vector with P19 of *Tomato bushy stunt virus* or HC-Pro of *Tobacco etch virus* to suppress post-transcriptional gene silencing increased GUS expression by 44 and 83%, respectively. A non-replicating vector containing a leader sequence from *Cowpea mosaic virus* (CPMV-*HT*) modified for enhanced translation led to 70% higher transient GUS expression than a control treatment. In hairy roots, a TRV vector capable of systemic movement increased GUS accumulation by 150-fold relative to the analogous PVX vector. Histochemical staining for GUS in TRV-infected hairy roots revealed the capacity for achieving even higher productivity per unit biomass.

**Conclusions:**

For the first time, replicating PVX vectors and a non-replicating CPMV-*HT* vector were successfully applied toward transient heterologous protein expression in cell suspensions. A replicating TRV vector achieved transient GUS expression levels in hairy roots more than an order of magnitude higher than the highest level previously reported with a viral vector delivered by *A. tumefaciens*.

## Background

While a plethora of viral vectors have recently been developed for agroinfiltration of plants [1-4], there are few reports of successful application of these vectors to *in vitro* systems [5]. Plant cell suspensions and hairy roots combine the advantages inherent to plants with the environmental control, scalability, short production cycles, and containment of tissues cultured in bioreactors. The controlled and aseptic nature of these *in vitro* systems makes them readily compliant with good manufacturing practice and suitable for the production of therapeutic proteins [6].

Plant cell suspensions are composed of individual and small aggregates of plant cells cultured in a liquid medium containing hormones that promote proliferation and de-differentiation. Cell suspensions are readily scalable volumetrically in bioreactors by principles already developed for microbial and mammalian cell cultures [7]. In fact, plant cell suspensions have been scaled up to 75,000 L for the production of valuable secondary metabolites [8]. They are also amenable to pilot-scale culture in low capital cost bioreactors [9].

Unlike plant cell suspensions, hairy root cultures consist of a tissue with multiple cell types. Hairy roots develop as the consequence of the transfer of genetic information from *Agrobacterium rhizogenes*, a gram-negative soil bacterium, to a host plant. This ‘natural’ process leads to the emergence of genetically transformed hairy roots at the site of infection on the plant. The hairy root phenotype is characterized by fast, hormone-independent growth, lack of geotropism, lateral branching and genetic stability [10]. Hairy root cultures are also scalable in bioreactors [11].

Both plants and plant tissue cultures are amenable to a rapid, scalable and low-cost gene delivery method using recombinant clones of *Agrobacterium tumefaciens*. When co-cultured with plant tissues *A. tumefaciens* can efficiently mediate the transfer of heterologous T-DNA to the nucleus of the plant cells. Transient expression of this episomal DNA results in a temporal peak in heterologous protein expression in a matter of days. Consequently, transient expression is suitable for manufacturing proteins that require very short lead times. For example, the first doses of a plant-made influenza vaccine can be produced within three weeks of the release of sequence information for new pandemic strains [12]. In contrast, months are required to generate and select productive transgenic cell lines which can accumulate mutations in the transgenes over time when maintained by serial subculture [13].

In the case of RNA viral vectors, *A. tumefaciens* delivers cDNA that is transcribed to the viral RNA genome in the nucleus and exported to the cytoplasm. Subsequent RNA replication cycles amplify both viral and heterologous genes. Concurrent with genome replication, viral polymerases also synthesize subgenomic RNAs that direct target protein translation. These double-stranded RNA intermediates can trigger post-transcriptional gene silencing (PTGS). Subsequently, homologous RNA transcripts may fail to accumulate due to sequence-specific targeting and destruction [14]. The 19-kDa gene product (P19) from *Tomato bushy stunt virus* is a potent suppressor of PTGS triggered by transient expression in plants [15].

PVX has a monopartite plus-sense RNA genome encoding five open reading frames (ORFs). The first ORF encodes the RNA-dependent RNA polymerase required for viral replication. The central three overlapping ORFs, known as the triple gene block (TGB), are required for local movement of the virus to neighboring cells. The final ORF encodes the PVX coat protein (CP) which is required for virion assembly, cell-to-cell infection via plasmodesmata, and systemic movement through the vasculature. The viral polymerase is translated directly from the viral RNA transcript and synthesizes the subgenomic plus-sense RNAs from which the other four ORFs are translated [16,17]. In plants PVX has been utilized as a full-length expression vector capable of infecting distal tissues [18-20] as well as a deleted vector lacking viral genes essential for local and systemic movement [21].

The TRV genome is divided into two plus-sense single-stranded RNAs that are separately encapsidated. RNA-1 encodes the viral proteins responsible for replication and movement, while RNA-2 encodes the viral coat protein and the transgene in place of two non-structural ORFs required for nematode transmission [22]. The smaller RNA-2 is nonessential for systemic infection of plants, such that it can be extensively modified without negatively affecting virus stability [23]. Tobraviruses such as TRV have adapted for efficient replication and movement in the roots of infected plants. Here they invade meristematic tissues, such as growing tips, to be ingested by specific nematode transmission vectors [24]. While replicating TRV and PVX vectors were reported to express GFP to similar levels in systemically infected leaves of *N. benthamiana,* infection with TRV resulted in 10 to 25-fold more GFP accumulation in the roots of these plants. This disparity in GFP expression was attributed to the root tropism of TRV [23].

While PVX and TRV vectors require viral replication to achieve high-level expression, the CPMV-*HT* expression system relies on enhanced translation in the absence of viral replication [1]. The pEAQ-HT vector based on this system includes the P19 gene on the same T-DNA as the target gene [25]. In a side-by-side comparison, the non-replicating ‘hyper-translatable’ CPMV-*HT* expression system yielded five times more anti-HIV monoclonal antibody than the analogous replicating deleted version of CPMV RNA-2, without detrimentally impacting post-translational modifications [26].

In this study we evaluated the three RNA viral vectors described above for their potential to achieve rapid, high-level *Agrobacterium*-mediated transient protein expression in *N. benthamiana* cell suspensions and hairy roots.

## Methods

### Plants and plant tissue cultures

Callus cultures of wild-type *N. benthamiana* were generated by placing leaf and petiole tissue explants from aseptically-grown seedlings onto MSG media solidified with 6 g/L agar. MSG is a derivative of MS salts media [27], containing 25 g/L sucrose, 1X B5 vitamins [28], 0.5 mg/L 2,4-dichlorophenoxyacetic acid (2,4-D), and 0.2 mg/L kinetin with the pH adjusted to 5.7. Following callus induction, liquid cell suspension cultures were subsequently maintained on MS media with 0.2 mg/L 2,4-D and 25 g/L sucrose pH adjusted to 5.5 in 500-mL Erlenmeyer flasks. Suspensions were serial subcultured every 14 days by transferring 20 mL into 100 mL of fresh media and returning 20 mL to the original flask to achieve a final volume of 100 mL. Callus generated from four individual plants was used to establish four cell lines consisting of fine liquid suspensions. Suspensions were screened by co-culture with *A. tumefaciens* harboring pBY031-I1. A single cell line was selected and used for subsequent experiments based on good growth and superior transient GUS expression after 4 days of co-culture as determined by the qualitative assay.

Hairy root cultures were generated by transforming aseptically grown *N. benthamiana* seedlings with *A. rhizogenes* [ATCC:15834]. Individual roots that grew from different wound sites exhibiting the hairy root phenotype of fast, highly-branched, and hormone-independent growth were cultured separately and cured of bacteria on B5 medium supplemented with 300 mg/L cefotaxime. A single root clone was selected based on good growth and used for subsequent experimental work. Hairy root cultures were maintained on B5 medium with 25 g/L sucrose pH adjusted to 5.5 in 125-mL Erlenmeyer flasks and were serial subcultured every 14 days by transferring ~500 mg into 50 mL of fresh media [28]. All cell suspensions and hairy root cultures were incubated at 25°C in the dark on a 120 RPM orbital shaker with a 2.5 cm stroke.

*Nicotiana benthamiana* plants were grown from seed under fluorescent lighting with a 16 hour photoperiod in an incubator maintained at 23°C. Plants were grown in 10 cm square pots with Miracle-Gro potting mix containing sphagnum peat moss and perlite in a 3:1 ratio supplemented with dolomitic lime at the recommended concentration of 5.3 g/dm^3^. Plants were fertilized with liquid Dyna-Gro 7-9-5 at each watering at the rate recommended for outdoor plants (0.0326% by volume).

### Co-culture of plant tissues with *Agrobacterium tumefaciens*

Plant cell suspensions were prepared for an experiment by adding 5 mL of 2-week-old suspension culture into 15 mL of MS media supplemented with 0.2 mg/L 2,4-D, 25 g/L sucrose and 2.5 mM KH_2_PO_4_ in a 125-mL flask with a loose foil closure and no sponge plug. Within two hours, *A. tumefaciens* clones harboring the appropriate plasmids were then added to initiate co-culture. Root cultures were initiated by adding ~500 mg fresh weight of 2-week-old roots into 25 mL of B5 media in a 125-mL flask with a loose foil closure. Unless noted otherwise, root cultures were incubated under standard conditions for 5 days prior to initiation of co-culture.

The *A. tumefaciens* strain used for co-culture with plant suspensions was the cysteine auxotroph C58::pEHA105/Cys32, which was generated by transposon mutagenesis [29]. Growth of the bacteria was monitored in liquid culture on the basis of measurements of absorbance at 600 nm (OD_600_). *A. tumefaciens* clones harboring the appropriate plasmids were cultured individually in Luria-Bertani (LB) media supplemented with 50 mg/L kanamycin in 25x150mm culture tubes on a 1.9 cm stroke rotary shaker at 250 RPM and at 25°C. Cryogenic stocks of each *Agrobacterium* clone were used to inoculate 2 mL LB media with selection. These cultures were then grown overnight and used to quantitatively inoculate 5 mL aliquots of fresh LB media with selection to achieve the desired bacterial density based on an additional 6 to 8 hours of growth. Once the *A. tumefaciens* cultures reached an exponential growth phase (OD_600_ ~1) they were pelleted by centrifugation at 3000 RCF for 7 min, washed, and resuspended in root or cell suspension medium to an OD_600_ of approximately 1.0. Each bacterial culture was added to the plant tissue culture flasks at the equivalent of 1% of the working culture volume at an OD_600_ of 1.0. In all experiments involving pTRV2-GUS, a separate *A. tumefaciens* clone harboring pTRV1 was always co-inoculated in a 1:1 ratio.

### Histochemical GUS detection and quantification

After six days of co-culture, GUS expression in each flask was evaluated qualitatively by histochemical staining with X-Gluc substrate [30]. Approximately 50 mg fresh weight of plant cell suspension filter cake was added to 75 μL of isotonic solution (6 g/L sodium chloride) in a flat-bottom 96-well microtiter plate. 100 mg fresh weight of roots (blotted dry) was collected from the outside edge of the root mat and transferred to a 1.5 mL microcentrifuge tube. Freshly harvested samples were incubated at room temperature with 150 μL (cells) or 200 μL (roots) of X-Gluc staining buffer (2 mM 5-bromo-4-chloro-3-indolyl β-D-glucuronic acid, 10 mM EDTA, 0.1% Triton X-100, 100 mM sodium phosphate buffer at pH 7.0, 0.5 mM potassium ferricyanide, 0.5 mM potassium ferrocyanide). 200 mg samples were also collected for the quantitative MUG assay and stored at −20°C prior to processing.

The yield of GUS was quantified through a fluorometric assay. Protein was extracted by the addition of extraction buffer (333 mg/mL silicon carbide, 50 mM sodium phosphate buffer at pH 7.0, 10 mM sodium EDTA, 0.1% Triton X-100, 0.1% sodium lauryl sulfate, 10 mM 2-mercaptoethanol) at 0.45 mL/g fresh weight roots or cells, or 2 mL/g fresh weight leaf tissue. Tissue was frozen in liquid nitrogen and homogenized with a sterile polypropylene mini-pestle (Sigma-Aldrich, part number Z359947) in a 1.5 mL microcentrifuge tube at ~60 RPM for 80 seconds. Homogenized samples were centrifuged at 16,000 RCF for 10 minutes at 4°C. The total soluble protein (TSP) in the supernatant was quantified using a modified Bradford assay with bovine serum albumin as the protein standard [31]. Samples were then diluted with extraction buffer (silicon carbide omitted). GUS activity was quantified from approximately 0.1 microgram of TSP per sample in a 96-well white opaque microtiter plate using a three-point kinetic assay with 0.2 mM 4-methylumbelliferyl β-D-glucuronide (MUG) as the substrate at 37°C [32]. Relative fluorescent measurements were converted into yield of GUS as a percent of total soluble protein based on assays of GUS type VII-A from *E. coli* (Sigma-Aldrich) as a standard.

### Leaf infiltrations

Transient expression assays were performed using 4–5 week old *N. benthamiana* plants. Cultures of *A. tumefaciens* were prepared for leaf infiltrations as described for co-culture with plant tissue cultures with the following differences: cultures were washed and resuspended to a final OD_600_ of 1.2 in MMA media pH adjusted to 5.6 (10 mM MES (2-[*N*-morpholino]ethanesulfonic acid), 10 mM magnesium chloride, 100 μM acetosyringone). The cultures were then incubated for 2 hours at 25°C and pressure infiltrated into the abaxial surfaces of intact *N. benthamiana* leaves using a needleless 3 mL plastic syringe. Agroinfiltration was performed on the distal parts of leaves in the 5^th^ to 7^th^ tiers counting down from the first fully emerged leaf. At least four leaves on different plants were infiltrated for each treatment. Each leaf was infiltrated bilaterally with two independently harvested samples. For co-transformation of two or more *A. tumefaciens* clones containing different constructs, equal volumes of the individual cultures each at an OD_600_ of 1.2 were mixed together or diluted with MMA such that the final concentration of each clone was constant across comparative treatments. Control infiltrations comprised a mixture of pPSP19, pRep110 and MMA in a 1:1:1 ratio, or pPSP19 and MMA in a 1:1 ratio. Leaves were harvested after 6 days of co-cultivation except for the treatments containing pRep110, which were harvested after 5 days to avoid necrosis appearing at later times. Samples were harvested from each infiltration using a 1.9 cm diameter sharpened steel punch. The weight of each leaf disk was recorded (approximately 60 mg) and stored at −20°C prior to quantitative GUS analysis.

### Vector construction

**Figure 1:**
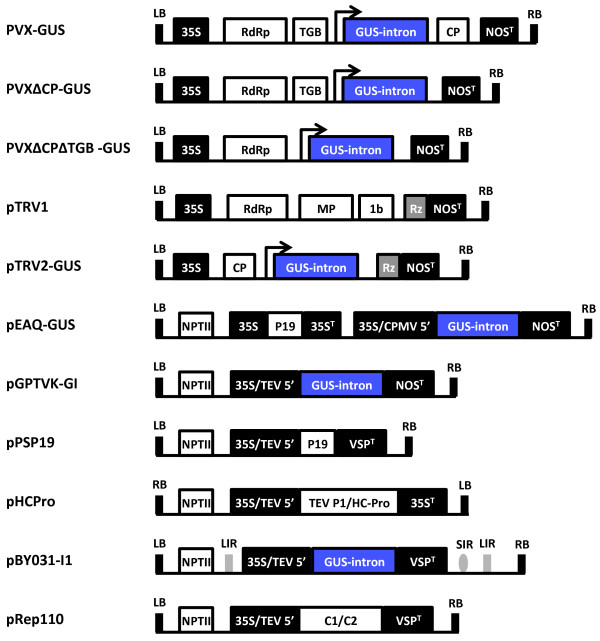
**Schematic representation of the vector T-DNA regions.** Control elements are illustrated in black boxes and coding sequences are shown in white or blue boxes. Overhead arrows represent duplicated subgenomic coat protein promoters driving expression of the inserted transgene. All vectors contain a backbone sequence derived from pBIN19 except for the PVX vectors, which are based on pGreen0000. 35S, dual enhanced *Cauliflower mosaic virus* (CaMV) 35S promoter; 35S^T^, CaMV 35S terminator; TEV 5’, *Tobacco etch virus* 5’ untranslated region; CPMV 5’, *Cowpea mosaic virus* 5’ untranslated region modified with mutations A115G and U162C; LB and RB, left and right T-DNA borders; RdRp, RNA-dependent RNA polymerase; TGB, triple gene block; CP, coat protein gene; Rz, self-cleaving ribozyme; NOS^T^, nopaline synthase terminator; MP, movement protein gene; VSP^T^, soybean vegetative storage protein B 3’ untranslated region; NPTII, expression cassette conferring kanamycin resistance; LIR and SIR, long and short intergenic regions of the *Bean yellow dwarf virus* genome; C1/C2, *Bean yellow dwarf virus* open reading frames encoding replication initiation proteins REP and REPA.

The constructs used in this study are summarized in Figure 1*.* The GUS reporter consists of the *uidA* gene modified by the insertion of the potato PIV2 intron to prevent bacterial expression as described previously for the construction of pBY031-I1 and pGPTVK-GI [5]. Binary vectors pPSP19 and pRep110 contain the *Tobacco bushy stunt virus* 19 kDa gene product (P19) and the native *Bean yellow dwarf virus* replication proteins, respectively [2]. Vector pHCPro contains the TEV P1/HC-Pro polyprotein as a HindIII fragment containing the dual 35S expression cassette from pRTL2-0027 in the pGA482 binary vector [33].

The vector pGR106 [GenBank:AY297843] consists of a full-length PVX cDNA containing a duplicated coat protein subgenomic promoter and multiple cloning site (MCS) upstream of the coat protein gene [34]. The 5’ end of the CP gene was removed from PGR106 by digestion with SalI and XhoI. Re-ligation of the compatible ends resulted in PVXΔCP, which retains a 61 bp 3’ terminal fragment of the CP important for viral replication [35]. Site-directed mutagenesis of PVXΔCP was used to introduce a 3^rd^ Bsu36I recognition site at the 3’ end of the triple gene block by removing the adenine residue at nucleotide 7647. A subsequent Bsu36I digestion and re-ligation yielded PVXΔCPΔTGB with a 1 kb deletion spanning the triple gene block. The GUS-intron coding sequence was PCR-amplified from pBY031-I1 using primers AscI_GI_F and GI_NotI_R (Table 1). The vector plasmids were sequentially digested with AscI and NotI while concurrently treated with shrimp alkaline phosphatase. The PCR-amplified insert was ligated into the AscI/NotI digested PVX vectors to create PVX-GUS, PVXΔCP-GUS and PVXΔCPΔTGB-GUS. Transformants were verified by colony PCR with primers Rep_F and GI_R. Amplified fragment sizes were 1.14 kb for PVXΔCPΔTGB-GUS and 2.13 kb for PVXΔCP-GUS and PVX-GUS. The integrity of the plasmids was further verified by dual restriction digest with AscI and PstI.

**Table 1:** Sense (F) and antisense (R) oligonucleotides used for PCR amplifications

**Primer**	**Sequence (5’ – 3’)**	**Hybridization site**
AscI_GI_F	**TAGGCGC**GCC*ATG*GTCCGTCCTGTAGAA	5’ terminus of GUS-intron
GI_NotI_R	**TTGCGGCCGC**AGAGGATCCTCATTGTTT	3’ terminus of GUS-intron
Rep_F	GCCTGAGTTTTGTGGTTGG	internal to PVX RdRp
GI_R	GGATAGTCTGCCAGTTCAGTTCG	internal to GUS-intron
GI_XhoI_R	**CCTACTCGAG**CCTCATTGTTTGCCTCCC	3’ terminus of GUS-intron
PEBVsgPR_F	AACTCGGTTTGCTGACCTAC	PEBV subgenomic promoter
TRV3UTR_R	ACCTAAAACTTCAGACACGG	3’ UTR of TRV RNA-2
AgeI-GI_F	**TATAACCGGT**C*ATG*GTCCGTCCTGTAGAAACC	5’ terminus of GUS-intron
pEAQ_F	AACGTTGTCAGATCGTGCTTCGGCACC	5’ UTR of pEAQ-*HT*
pEAQ_R	CTCCTGTTTAGCAGGTCGTCCCTTCAG	3’ UTR of pEAQ-*HT*

Sequences added with 5’ extensions are shown in bold with restriction enzyme recognition sites underlined. Initiation codons are italicized. UTR, untranslated region; PEBV, *Pea early browning virus*; RdRp, RNA-dependent RNA polymerase.

Binary vectors containing the full-length cDNA of TRV isolate PpK20 RNA-1 (pTRV1) and RNA-2 (pYL156-GFP) were described previously [36,37]. The GUS-intron coding sequence with flanking NcoI and XhoI restriction was generated by PCR amplification of pBY031-I1 with primers Asc_GI_F and GI_XhoI_R. The corresponding NcoI-XhoI fragment containing the GFP ORF in pYL156-GFP was digested out and replaced with the GUS-intron fragment to create pTRV2-GUS. An undocumented NcoI recognition site present in the pYL156 vector backbone sequence necessitated a triple ligation due to the generation of two backbone fragments. Transformants were PCR-verified with primers PEBVsgPR_F and TRV3UTR_R. The integrity of the plasmid was further verified by restriction digest with SphI. The entire transcribed region of pTRV2-GUS including the 5’ and 3’ untranslated regions was verified by sequencing.

To create pEAQ-GUS the GUS-intron coding sequence was PCR amplified from pBY031-I1 with primers AgeI_GI_F and GI_XhoI_R. This fragment was then cloned into the AgeI and XhoI sites of the pEAQ-*HT* MCS such that the optional histidine tags were replaced. Transformants were PCR-verified with primers pEAQ_F and pEAQ_R to amplify a 2.56 kb fragment.

The high fidelity polymerases Phusion (New England Biolabs) or iProof (Bio-Rad) were used according to the protocols suggested by the manufacturer to generate all cloning insert fragments. Ligation products were drop-dialyzed on 13 mm diameter 0.025 μm pore-size microdialysis membranes (MF-Millipore product code VSWP) prior to transformation into electrocompetent *E. coli* strains DH5α or DH10β [38]. Electrocompetent *E. coli* and *A. tumefaceins* were prepared and transformed using the Bio-Rad MicroPulser Electroporation Apparatus as per the manufacturer’s protocols. Plasmid pGR106 and its derivatives were co-transformed with the pSoup helper plasmid [39]. All *A. tumefaciens* and *E. coli* transformants were selected on LB medium supplemented with 50 mg/L kanamycin and incubated at 25°C and 37°C, respectively. All *A. tumefaciens* transformants were colony purified and subsequently verified by colony PCR.

### Statistical analysis

All probabilities were calculated using Welch’s *t* test with a two-tailed distribution assuming unequal variance between data sets consisting of at least three replicates.

## Results

### In cell suspensions, a replicating PVX vector yielded 6.6-fold more GUS when non-essential viral genes were omitted

In the present study three different PVX constructs were characterized: a PVX full-length vector containing GUS as a gene insert (PVX-GUS) and derivatives of that vector containing deletions spanning most of the coat protein (PVXΔCP-GUS) or both the coat protein and triple gene block (PVXΔCPΔTGB-GUS). The latter vector is referred to as a minimal vector since it retains only the viral components required for replication, including the RNA-dependent RNA polymerase. In all cases a plant intron within the GUS gene prevents bacterial expression. GUS production by *A. tumefaciens* has been demonstrated from a deleted vector of *Foxtail mosaic virus,* a potexvirus closely related to PVX [40].

**Figure 2:**
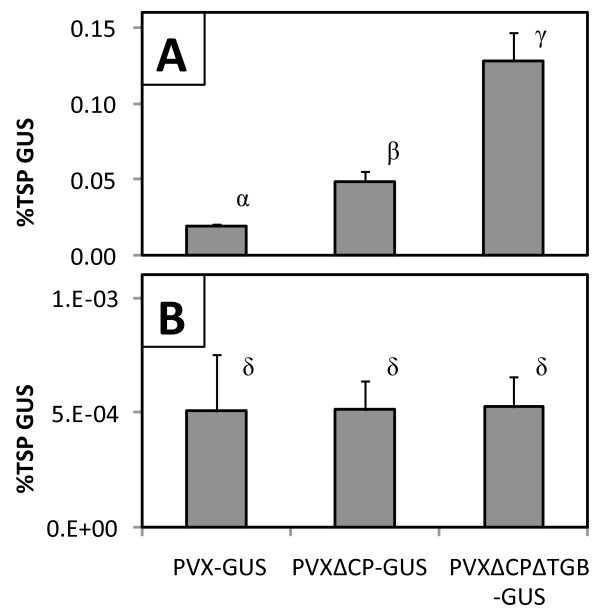
**Transient GUS expression from PVX vectors in cell suspensions and hairy roots.** The PVX-GUS vector containing GUS as an insert driven by a duplicated coat protein subgenomic promoter was compared to a coat protein replacement construct (PVX∆CP-GUS) and a minimal vector lacking the coat protein and the triple gene block (PVX∆CP∆TGB-GUS) in (**A**) cell suspensions, and (**B**) hairy roots. Values represent the mean ± SD of samples from three co-cultures. Means labeled with the same Greek letter are not different from each other at the 95% significance level.

In cell suspensions the PVX movement functions encoded by the TGB and CP genes were dispensable when complemented by the efficient viral cDNA delivery capabilities of *A. tumefaciens*. Transient GUS expression was 6.6-fold higher with the minimal vector relative to the full-length vector (Figure 2A). Expression from the CP-replacement vector was intermediate between the full-length and minimal vectors and significantly different from both. Thus, vectors lacking non-essential viral genes were free to direct more of the cellular metabolic resources towards the production of the target protein. Relative to cell suspensions, average transient GUS expression from the minimal PVX vector was 244-fold lower in hairy roots, where the presence of the CP and TGB genes had an insignificant effect (Figure 2B).

The relative performance of the PVX vectors in cell suspensions is consistent with previous reports of similar vectors in leaves. Omission of the PVX CP gene led to higher target protein expression in infiltrated *N. benthamiana* leaves [41]. Similarly, a minimal PVX vector (PVXdt-GFP) lacking both the CP and TGB genes yielded 2.5-fold more transiently expressed GFP relative to a full-length vector when co-expressed with HC-Pro [35].

### Suppression of post-transcriptional gene silencing increased transient GUS expression from the minimal PVX vector in cell suspensions

**Figure 3:**
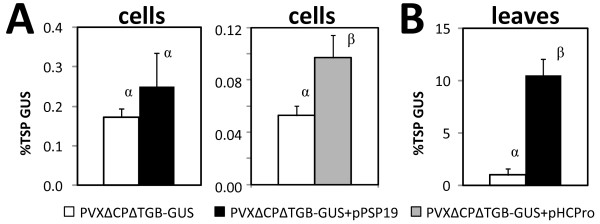
**Suppression of post-transcriptional gene silencing in cell suspensions and leaves.** The minimal PXV vector (PVX∆CP∆TGB-GUS) was transiently transformed alone or co-transformed with a suppressor of PTGS (either pPSP19 or pHCPro). Values represent the mean ± SD of samples from (**A**) three cell suspension co-cultures, and (**B**) three infiltrated leaves of intact plants. Means on the same axis labeled with the same Greek letter are not different from each other at the 95% significance level.

**Figure 4:**
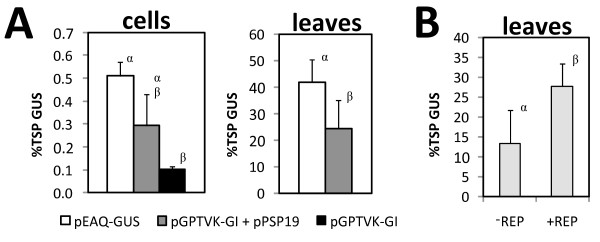
**Transient GUS expression from non-replicating and replicating viral vectors in cell suspensions and leaves.** (**A**) Non-replicating vectors contained the GUS-intron gene downstream of a conventional TEV 5’UTR (pGPTVK-GI) or a modified CPMV 5’ UTR (pEAQ-GUS). The latter vector provided P19 as a second expression cassette on a single T-DNA. Values represent the mean ± SD of samples from three co-cultures or four infiltrated leaves. (**B**) The replication-competent vector pBY031-I1 and pPSP19 were co-delivered to *N. benthamiana* leaves together (−REP) or with a third binary vector containing the *Bean yellow dwarf virus* DNA replicase gene (+REP). Values represent the mean ± SD of samples from seven leaf infiltrations on different leaves. Means on the same axis labeled with the same Greek letter are not different from each other at the 95% significance level.

The minimal PVX vector contains a deletion in the triple gene block that includes the P25 gene encoding the native suppressor of PTGS [42]. Thus, we sought to potentially complement this disrupted function through co-expression of P19. Co-transformation of the minimal vector (PVXΔCPΔTGB-GUS) with pPSP19 increased transient GUS expression by 44% and 990% in co-cultured cell suspensions (Figure 3A) and infiltrated leaves (Figure 3B), respectively. This increase was statistically significant in leaves but not cell suspensions. A similar trend was observed for transient co-expression of the HC-Pro suppressor of *Tobacco etch virus,* which led to a statistically significant 83% increase in transient GUS expression in cell suspensions (p < 0.05; Figure 3A). Co-transformation with pHCPro also increased transient GUS expression in leaves, but severe necrosis present at six days post-infiltration precluded accurate quantification. Similar results were observed with a non-replicating expression vector: Co-transformation of pGPTVK-GI with pPSP19 increased transient GUS expression by 184% in cell suspensions (Figure 4A).

Consistent with the results of the present study, *N. benthamiana* leaves infiltrated with PVXdt-GFP accumulated more GFP when HC-Pro was co-expressed [35]. Similarly, co-transformation of a non-replicating conventional vector with a P19 construct significantly increased peak transient expression of an IgG antibody by 75% and 1300% in co-cultured *N. benthamiana* cell suspensions and infiltrated leaves, respectively [43].

### A non-replicating vector containing a leader sequence from *Cowpea mosaic virus* modified for enhanced translation led to higher transient GUS expression in cell suspensions

Relative to the control treatment, pEAQ-GUS resulted in approximately 70% higher transient GUS expression in both co-cultured cell suspensions and infiltrated leaves (Figure 4A). The increase in expression was statistically significant in leaves but not cell suspensions. Average transient GUS expression from the pEAQ-GUS vector in cell suspensions (0.51% TSP) was consistent with the only other report pertaining to the use of the CPMV-*HT* expression system in this host. Tobacco Bright Yellow-2 cells stably transformed with pEAQ-SP-*HSA* expressed recombinant human serum albumin (rHSA) at 11.88 mg/L in the culture medium, accounting for 0.7% TSP. In contrast a conventional CaMV 35S-based vector (lacking P19) failed to yield detectable quantities of secreted rHSA [44].

### A TRV vector capable of systemic movement outperformed the analogous PVX vector in hairy roots

**Figure 5:**
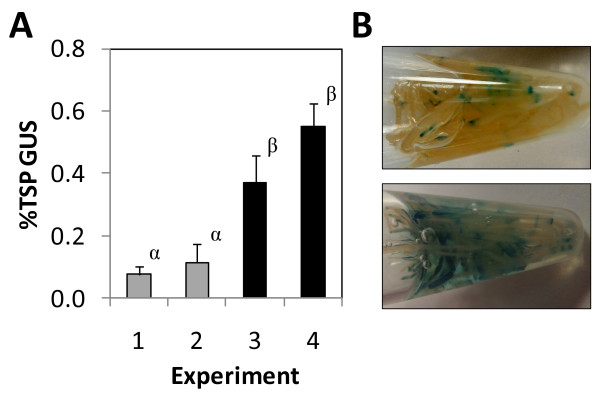
**Transient GUS expression from a TRV vector in hairy roots.** (**A**) Hairy root cultures were initiated and incubated for 5 (gray bars) or 7 days (black bars) prior to co-culture with *A. tumefaciens* clones harboring pTRV1 and pTRV2-GUS. Values represent the mean ± SD of three co-cultures harvested six days after initiation. Four separate experiments were conducted over a period of 13 months. Means labeled with the same Greek letter are not different from each other at the 95% significance level. (**B**) Histochemical detection of transient GUS expression in representative samples collected from Experiments 1 (top) and 4 (bottom). Hairy roots were incubated with X-Gluc substrate in 1.5 mL microcentrifuge tubes for 60 minutes at room temperature. Areas stained blue are positive for GUS expression.

The pTRV2-GUS vector yielded 150-fold more GUS than PVX-GUS (Table 2). Thus the results of the present study extend the root tropism of TRV to *in vitro* cultures of hairy roots. In fact, all other vectors tested to date have proven to be unsuitable for high-level transient expression: Hairy roots infected with the TRV vector expressed at least an order of magnitude more GUS than the highest level previously reported with the replicating pBY031-I1 vector derived from *Bean yellow dwarf virus*. Thus TRV yielded transient expression levels in hairy roots that are on par with those previously observed only in plant cell suspensions, which typically exceed levels in hairy roots by 20-fold for vectors lacking viral movement functions [5]. Even more remarkable is that this magnitude of expression reflected that obtained from a non-optimized process. Simply initiating the co-culture 7 rather than 5 days after hairy root culture initiation significantly increased average transient GUS expression by more than 4-fold to 0.5% TSP (Figure 5A).

**Table 2:** Comparison of different viral vectors used for transient GUS expression in hairy roots

**Construct**	**Species**	**GUS (%TSP)**	**Std. dev.**	**Days in co-culture**
pTRV2-GUS	*N. benthamiana*	0.076	0.027	6
PVX-GUS	*N. benthamiana*	0.0005	0.0002	6
pEAQ-GUS	*N. benthamiana*	0.006	0.004	4
pBY031-I1	*N. glutinosa*	0.007	0.003	3

### Transient GUS expression in hairy roots underestimates the capacity for heterologous protein production

For a movement-enabled vector such as TRV, transient GUS expression is expected in both cells directly colonized and transiently transformed by *A. tumefaciens* as well as distal cells susceptible to secondary infection. Primary infection occurs in actively dividing surface cells that are accessible by *A. tumefaciens* and amenable to T-DNA transfer such as adventitious laterals, root tips and the root zone of elongation [5]. Cell-to-cell and systemic viral movement lead to secondary infections that occur over a period of days following primary infection.

Histochemical staining of hairy roots co-cultured with pTRV2-GUS revealed a heterogeneous pattern of GUS expression (Figure 5B). Heterogeneous expression implies that there is room for improving productivity per unit biomass because bulk samples are diluted by non-transformed tissue. Thus the reported expression levels do not reflect the capacity for expression on the cellular level. Simply extending the duration of the co-culture did not increase qualitative GUS expression, suggesting that the kinetics of viral movement were not limiting (results not shown). Instead, it appears that this combination of viral vector and host tissue may be suboptimal.

## Discussion

There have been several reports of RNA viruses engineered for high-level heterologous protein expression in cell suspensions and hairy roots. The highest reported level of heterologous protein expression from a viral vector in plant tissue culture was achieved with an estrogen-inducible promoter fused to the cDNA of a *Tomato mosaic virus* CP-replacement vector. A tobacco BY-2 cell suspension stably transformed with this vector accumulated GFP to 10% TSP [45]. A full-length *Tobacco mosaic virus* (TMV) vector has also been used to transiently express GFP in *N. benthamiana* cell suspensions and hairy roots co-incubated with infectious recombinant viral particles. The recombinant virus failed to replicate or lead to significant GFP accumulation in hairy roots due to foreign gene deletion that occurred during serial passaging of the recombinant TMV stocks in plants [46]. Similarly, very limited uptake and replication was observed for infectious recombinant TMV particles co-incubated with cell suspensions [47]. In an alternative approach, hairy roots of *N. benthamiana* were established from the leaves of plants previously infected with the same recombinant TMV vector. These clonal hairy root lines sustained recombinant virus replication and GFP expression over a period of three years in the absence of selection at levels comparable to those found in transgenic hairy roots [48].

In contrast, our approach of harnessing an engineered strain of *A. tumefaciens* to amplify and deliver viral cDNA to pre-established plant tissue cultures avoids the instability and long lead times associated with viral passaging in whole plants or establishing recombinant virus-infected root lines from infected leaves. Moreover, viral cDNA transfection by *A. tumefaciens* in plant tissue cultures may be more efficient and reproducible than passive uptake of viral particles. Agroinfiltration led to almost complete infection in leaves even with TMV viral vectors disabled for cell-to-cell movement [49].

The relatively low expression we consistently observed in cell suspensions (approximately 0.5% TSP, or 3 mg/L) was not the result of incompatibilities of the viral vectors with this host (*N. benthamiana*). Viral vectors of disparate origin that rely on three distinct mechanisms of transgene amplification (DNA replication for *Bean yellow dwarf virus*, mRNA replication for PVX, and hyper-translation for CPMV-*HT*) demonstrated high-level expression in leaves, but not cell suspensions. For example, using a DNA viral vector derived from *Bean yellow dwarf virus,* transient GUS expression was 125-folder higher in *N. benthamiana* leaves (27.6% TSP; Figure 4B) relative to *N. glutinosa* cells (0.22% TSP; [5]). In both hosts complementation of pBY031-I1 with the viral replicase protein-supplying vector (pRep110) had the effect of doubling relative GUS expression levels, demonstrating functional viral amplification abilities. Moreover, pGPTVK-GI, pEAQ-GUS and PVX∆CP∆TGB-GUS yielded 70 to 80-fold more GUS in leaves relative to cells in the presence of P19. Likewise, transient co-expression of a murine IgG1 with P19 was reported to be 75-fold higher in infiltrated *N. benthamiana* leaves relative to cell suspensions [43]. These results are inconsistent with the commonly held notion that the application of viral vectors and/or suppression of gene silencing may readily overcome the low titers characteristic of transient expression in cell suspensions.

The unique physiology of cell suspensions may account for the observed lower absolute expression levels that were recalcitrant to suppression of post-transcriptional gene silencing. Relative to cell suspensions, mature leaves may provide a more natural environment for the attachment of *A. tumefaciens* and subsequent transfer of T-DNA. Yet, the highest yields for transient expression have been achieved in heterotrophic plant cell suspensions undergoing rapid cell division, whereas non-dividing but metabolically active cells in fully expanded leaves tend to work best *in planta*[50,51]. Consequently, differences in physiological state other than cell cycle status must play an important role in mediating transient protein expression of ectopic DNA.

Plant cell suspensions are composed of individual or small aggregates of dedifferentiated cells that lack fully functional plasmodesmata due to minimal cell-cell contacts. Hence, systemic PTGS may be reduced since the silencing signal is generally transmitted via plasmodesmata and the vascular system [52,53]. Protein degradation in cell suspensions has been recognized to play an important role in limiting overall heterologous protein yields to less than 1% TSP [54]. The intracellular concentration of a protein reflects a balance between its rate of synthesis and degradation. Thus, strategies to amplify protein synthesis through upstream processes including transcription and translation may be negated or obscured by the effect of increased protein turnover. Moreover, this protein degradation is likely non-specific since GUS is highly stable within the cytoplasmic environment and has even been used as a protein-stabilizing fusion partner [55,56]. Co-expression of a heterologous protease inhibitor represents a potential method to down-regulate protein turnover and increase the accumulation of target protein in cell suspensions [57].

## Conclusions

To the best of the authors’ knowledge this is the first report of the application of RNA viral vectors for *Agrobacterium*-mediated transient protein expression in plant tissue culture. Furthermore, the TRV vector described here represents the first and only vector to achieve transient expression levels in hairy roots comparable to those observed in plant cell suspensions using this method. Despite equivalent or superior performance in side-by-side comparisons with non-viral vectors in plant cell suspensions, the PVX and CPMV-*HT* vectors did not overcome the batch-to-batch variability that characterizes transient protein expression in this platform. However, the reported trends were reproducible across independent experiments notwithstanding these fluctuations in absolute expression levels.

Our future work will focus on engineering hairy root lines with integrated suppression of PTGS and developing TRV vectors with increased infectivity. Hairy roots transformed to constitutively express a strong suppressor of PTGS may support higher levels of transient protein expression because the native 16 kDa gene product of TRV is relatively weak compared to P19 of *Tomato bushy stunt virus*[58]. Optimization of a TMV vector by removal of putative splice sites and insertion of multiple plant introns increased the efficiency of viral replication initiation by up to three orders of magnitude in *N. benthamiana* infiltrated leaves [49]. Additionally, a modified TRV expression vector retaining the native 2b gene on RNA-2, required for nematode transmission, displayed increased infectivity and root meristem invasion in *N. benthamiana* plants [24]. Similar modifications to pTRV1 and pTRV2-GUS could improve the efficiency of primary (nuclear-launched) infection and systemic movement. We anticipate that the implementation of these improvements to this vector and host combination will yield the high and homogeneous expression characteristic of infiltrated leaves.

## Abbreviations

PVX: Potato virus X; TRV: Tobacco rattle virus; GUS: β-glucuronidase; CPMV: Cowpea mosaic virus; PTGS: Post-transcriptional gene silencing; ORF: Open reading frame; TGB: Triple gene block; CP: Coat protein; TSP: Total soluble protein; TEV: Tobacco etch virus; MCS: Multiple cloning site; GFP: Green fluorescent protein; TMV: Tobacco mosaic virus.

## Competing interests

The authors declare that they have no competing interests.

## Authors' contributions

JSL was responsible for experiment design, execution, analysis and interpretation, and wrote the manuscript. WRC conceived the study, oversaw the experimental work, contributed to interpretation, and edited the manuscript. Both authors read and approved the final manuscript.
